# Synthesis and structure–activity relationships for some novel diflapolin derivatives with benzimidazole subunit

**DOI:** 10.1080/14756366.2022.2087645

**Published:** 2022-09-20

**Authors:** Lisa Vieider, Eva Zoeller, Erik Romp, Martin Schoenthaler, Victor Hernández-Olmos, Veronika Temml, Thomas Hasenoehrl, Daniela Schuster, Oliver Werz, Ulrike Garscha, Barbara Matuszczak

**Affiliations:** a Department of Pharmaceutical Chemistry, Center for Chemistry and Biomedicine, Institute of Pharmacy, University of Innsbruck, Innsbruck, Austria; b Department of Pharmaceutical/Medicinal Chemistry, Institute of Pharmacy, University of Jena, Jena, Germany; c Branch for Translation Medicine and Pharmacology TMP, Frauenhofer Institute for Molecular Biology and Applied Ecology IME, Frankfurt am Main, Germany; d Department of Pharmaceutical and Medicinal Chemistry, Institute of Pharmacy, Paracelsus Medical University Salzburg, Salzburg, Austria; e Department of Pharmaceutical/Medicinal Chemistry, Institute of Pharmacy, University of Greifswald, Greifswald, Germany

**Keywords:** Diflapolin, benzimidazoles, soluble epoxide hydrolase (sEH), 5-lipoxygenase activating protein (FLAP), solubility and GI absorption prediction

## Abstract

A series of derivatives of the potent dual soluble epoxide hydrolase (sEH)/5-lipoxygenase-activating protein (FLAP) inhibitor diflapolin was designed, synthesised, and characterised. These novel compounds, which contain a benzimidazole subunit were evaluated for their inhibitory activity against sEH and FLAP. Molecular modelling tools were applied to analyse structure–activity relationships (SAR) on both targets and to predict solubility and gastrointestinal (GI) absorption. The most promising dual inhibitors of these series are **5a**, **6b,** and **6c**.

## 1. Introduction

**Figure 1.**
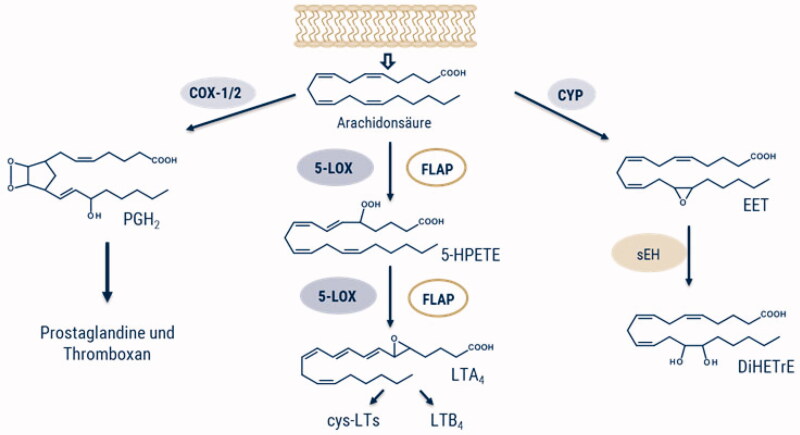
Overview of the arachidonic acid cascade with its major metabolic pathways and possible pharmacological targets.

A wide range of chronic pathologies (e.g. rheumatoid arthritis and asthma bronchiale) are related to inflammation.^1^ Centre of the inflammation process is the arachidonic acid (AA) cascade. Through activation of phospholipases, especially the cytosolic phospholipase A_2_α (cPLA_2_α),^2^ AA is released from cellular membranes. Different fates for metabolism of free AA into pro-inflammatory (Figure 1), as well as specialised pro-resolving lipid mediators^3^ are known.

The cyclooxygenase (COX) branch has been studied extensively over the last decades, and especially COX inhibitors are standard treatment for acute inflammatory conditions. However, in long-term use they cause gastrointestinal and cardiovascular side effects.^4^^,^^5^ Due to the importance of inflammation in chronic pathologies, targets within the other two branches of the pathway are shifting into focus.^6^ The metabolic pathways of cytochrome (CYP) P450 and 5-lipoxygenase (5-LOX) are currently of great interest to develop new anti-inflammatory drugs with less harmful side effects.

Already known, leukotrienes (LTs) are biosynthesised via the 5-LOX pathway to play a key role in the innate immune response as potent chemoattractant. However, ongoing LT biosynthesis is associated to chronic inflammatory processes. 5-LOX dioxygenases AA to hydroperoxyeicosatetraenoic acid (5-HPETE) and subsequently to leukotriene A_4_ (LTA_4_),^7^ the precursor for LTB_4_ and cysteinyl LTs formation.^8^ Interestingly, cellular LT biosynthesis is dependent on a nuclear membrane protein, the 5-LOX-activating protein (FLAP) that forms an essential biosynthetic complex with 5-LOX and promotes LTA_4_ formation.^9^^,^^10^

To prevent the leukotriene biosynthesis and therefore significantly intervene with the inflammatory process, there are two possibilities up to the current scientific level, (i) direct inhibition of 5-LOX for example by zileuton^11^^,^^12^ or (ii) prevention of the 5-LOX/FLAP complex formation by FLAP inhibitors as MK-886.^9^ Research has shown that direct 5-LOX inhibition can be achieved using three different methods (redox active compounds, non-redox types, and iron-ligands),^13^ but to date only zileuton has received approval. According to the latest information, it has been possible to synthesise zileuton-hydroxycinnamic acid hybrids, of which one candidate shows very promising inhibitory properties, being up to three times as active as its precursor, zileuton.^12^ However the hope are on FLAP inhibitors, some of them are currently undergoing clinical trials (i.e. GSK-2190915 by Amira Pharmaceuticals and GlaxoSmithKline completed clinical phase II studies for treatment of asthma patients; licofelone by Merkle, Alfa Wassermann, and Lacer reached clinical phase III for the treatment of knee osteoarthritis [ClinicalTrials.gov]). Beside the COX and LOX pathway, AA is also metabolised by cytochrome P450 (CYP), which leads to the formation of epoxyeicosatrienoic acids (EETs).^14^ Those epoxygenases introduce an oxygen atom on a carbon atom of the double bonds and regioisomers of EETs can arise. The latter have very positive properties, they can prevent the activation of nuclear factor kappa B (NF-κB),^15^ among other things, cardioprotective, anti-inflammatory effects on vascular cells such as endothelial cells and monocytes were found. The inhibition of pro-inflammatory mediators and cell adhesion molecules should also be noted.^16^ Unfortunately, EETs undergo a rapid cellular reduction to the corresponding polar and less active dihydroxyeicosatrienoic acids (DHETrEs) via soluble epoxide hydrolase (sEH).^14^ Inhibition of sEH is therefore a promising strategy to intervene with the inflammatory cascade. The EETs with anti-inflammatory properties are retained, further metabolism and hence the formation of pro-inflammatory mediators is also prevented.

Moreover, it is well-known that crosstalk between the branches of AA cascade occurs. This crosstalk is especially evident when trying to inhibit one single branch.^1^ One of the early pieces of evidence on this subject is aspirin-induced asthma. It was discussed that the reaction is due to COX inhibition, since the free AA was shifted over to the 5-LOX pathway and converted to leukotrienes. ^17^ It should be mentioned that the system of crosstalk is not quite as easy to explain as that the AA strolls back and forth within the pathways. This inter-pathway crosstalk has not yet been sufficiently investigated and needs more detailed examination.^18^ Up to now, in most cases a single target has always been used to combat a certain disease. But in situations such as within the AA cascade, where it seems that one target alone cannot achieve much, the topic of polypharmacology comes into play. The advantage of multi target drugs (or designed multiple ligands DMLs) lies in the treatment of complex diseases such as inflammation, metabolic syndrome, and cancer. The possibility of reaching several targets with just one drug reduces the necessary dose and, as a result, fewer off-target side effects are to be expected.^18^ The number of FDA approved drugs in recent years shows the importance of this new approach for drug development. Below are some important examples of dual inhibitors within the AA cascade: a promising candidate is PTUPB, a dual sEH/COX-2 inhibitor—currently being studied in detail for bleomycin-induced pulmonary fibrosis.^19^ There are also various 5-LOX/sEH inhibitors, most of which were discovered via in-silico methods and are currently subject to further structural modifications. For example, the dual inhibitor KM55 inhibited the adhesion of leukocytes to endothelial cells by disturbing the leukocyte function.^20^

**Figure 2.**
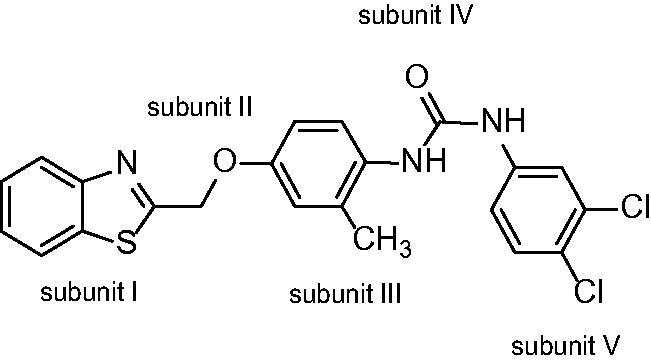
Diflapolin, a dual sEH/FLAP inhibitor divided into his five substructures.

Another new promising candidate for dual inhibition, diflapolin (Figure 2), was found in a pharmacophore-based virtual screening approach. Diflapolin acts as potent dual FLAP/sEH inhibitor with high target specificity. The compound shows no acute cytotoxicity, and it efficiently inhibits LT biosynthesis in vivo, combined with a strong anti-inflammatory activity in mouse.^21–23^

**Figure 3.**
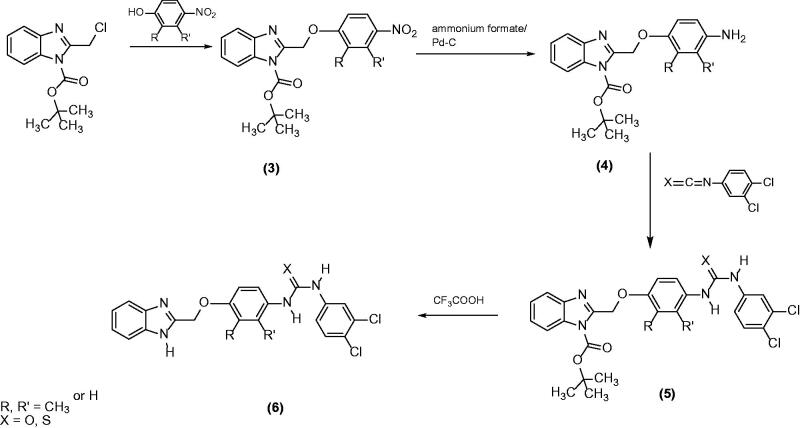
Synthesis of the novel diflapolin derivatives.

**Figure 4.**
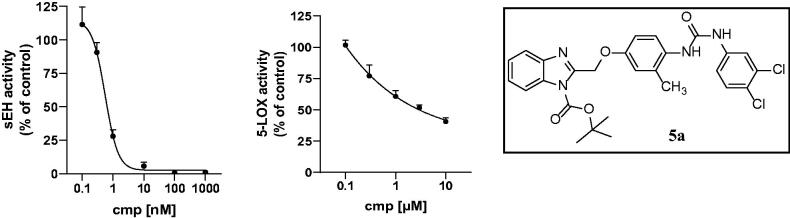
IC_50_ values of compound **5a**, sEH 0.7 ± 0.1 nM, FLAP 3.7 ± 0.9 µM.

**Figure 5.**
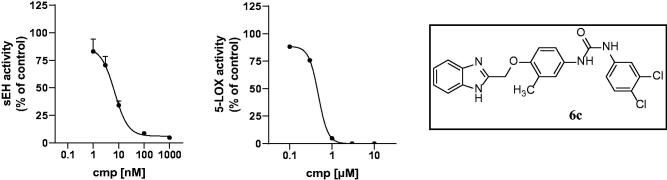
IC_50_ values of compound **6c**, sEH 4.9 ± 1.0 nM, FLAP 0.4 ± 0.01 µM.

### 1.1. Aim of the study

Structure modifications described in this work attend to provide a more precise insight into the structure–activity relationship of diflapolin. Within this study, we aimed to continuously improve the bioavailability, the selectivity, and the biological activity. The lead compound diflapolin shows poor solubility. The main reason for that is the *N,N’*-diaromatic substituted urea function, but which is also essential for sEH inhibition. Therefore, we modified diflapolin mainly apart from the urea moiety but with the aim to increase the solubility and retain bioactivity.

All potent sEH inhibitors have as common structural feature a 1,3-disubstituted urea, carbamate, or amide and FLAP inhibitors often consist of a bicyclic unit. In initial optimisation efforts, we moderately modified the lead compound diflapolin and altered exclusively the methyl substituted para-phenylene spacer (subunit III) and the terminal phenyl moiety (subunit V). The investigations showed that compounds with a methyl group close to the urea structure show good or better activity than diflapolin. However, compounds without a methyl group or with the methyl group near the benzothiazole core are biologically active too. At the terminal phenyl ring, it has been found that a 3,4-dichloro substitution, as can be found also in the lead structure, has the best inhibitory potencies.^24^

In the present study, modifications were carried out to the benzothiazole core (subunit I). With the exchange of the benzothiazole by a benzimidazole, we aimed to elucidate whether a benzothiazole skeleton is essential for FLAP and sEH inhibition and possibly improve solubility. To determine the structure–activity relationship of the benzimidazole together with the substituted para-phenylene spacer (subunit III), we synthesised a series of analogues with and without a methyl moiety. Furthermore, we aimed to modify the urea substructure (subunit IV) and therefore synthesised the corresponding products with a thiourea moiety in order to identify the need of the oxygen for the binding pocket. The favoured 3,4-dichloro substitution on the phenyl ring (subunit V) was retained.

## 2. Results and discussion

### 2.1. Chemistry

For preparation of the compounds with the benzimidazole core, the synthetic strategy developed in our group^21^^,^^24^ was slightly modified (see Figure 3). Starting from 2-chloromethylbenzimidazole **(1)**, the *tert*-butoxycarbonyl group was introduced by reaction with di-*tert*-butyl dicarbonate. This *N*-substituent served on the one hand as a protecting group^25^ for the nitrogen and on the other hand as a further structural modification. The reaction of the resulting 1‐*tert*‐butoxycarbonyl‐2-chloromethylbenzimidazole **(2)** with the methyl substituted or unsubstituted 4-nitrophenol in the presence of base (potassium carbonate) afforded compounds **3a–c**. Subsequent reduction of the nitro function with ammonium formate and palladium on charcoal as catalyst in methanol led to the appropriate amines **4a–c**. The first target compounds with *N*-Boc substituent (i.e. **5a–f**) became accessible by reaction of the amines with 3,4-dichlorophenylisocyanate or 3,4-dichlorophenylisothiocyanate, respectively. Subsequently, the *N*-substituent was split off with trifluoroacetic acid, which led to the second type of target compounds **6a–f**.

### 2.2. Biological evaluation

The effect of all novel urea and thiourea derivatives on the sEH and FLAP-dependent 5-LOX product formation was studied.

All diflapolin derivatives were tested for their inhibitory potency against sEH and FLAP in cell-free and cellular assays, respectively. IC_50_ values (see Table 1 and Figures 4 and 5) were determined for both assays using GraphPad Prism 8 software (Graphpad Software Inc., San Diego, CA, USA).

**Table 1.** Overview of the IC_50_ values of the newly synthesised diflapolin derivatives and state-of-the-art inhibitors for sEH and FLAP.

Compound	R	R′	X	FLAP (5-LOX product formation) IC_50_ ± SEM (µM)	she IC_50_ ± SEM (nM)
**Diflapolin**				neutrophils: 0.17 µM^23^	20 nM^23^
**AUDA (reference inhibitor of sEH)**					69 nM^26^
**MK-886 (reference inhibitor of FLAP)**				neutrophils: 0.01–0.014 µM ^27^	
**5a**	H	Me	O	3.7 ± 0.9	0.7 ± 0.1
**5b**	H	H	O	>10 µM	0.63 ± 0.1
**5c**	Me	H	O	>10 µM	0.7 ± 0.1
**5d**	H	Me	S	>10 µM	>10 µM
**5e**	H	H	S	>10 µM	19.1 ± 7.2
**5f**	Me	H	S	>10 µM	17.5 ± 12.7
**6a**	H	Me	O	0.7 ± 0.1	56.9 ± 19.4
**6b**	H	H	O	0.6 ± 0.1	10.5 ± 1.9
**6c**	Me	H	O	0.4 ± 0.01	4.9 ± 1.0
**6d**	H	Me	S	0.6 ± 0.1	223.4 ± 85.8
**6e**	H	H	S	0.5 ± 0.1	270.9 ± 58.4
**6f**	Me	H	S	0.75 ± 0.17	771.6 ± 169.2

The following SAR regarding FLAP-mediated 5-LOX product formation in human neutrophils was observed: for subunit I, *N*-butoxycarbonyl-substitution of the benzimidazole is disadvantageous. All compounds lost their inhibitory potency against FLAP, apart from **5a**, but which is also significantly less active than its *N*-unsubstituted benzimidazole analogue **6a**. In the Free-Wilson analysis, this modification was assigned a high negative contribution to the overall activity (−0.778). Removing the *N*-substitution leads to a significant increase in FLAP activity. The unsubstituted benzimidazole compounds exhibit IC_50_ values in the sub-micromolar range and are almost as active as the lead compound diflapolin. For sEH inhibition, the opposite effect was observed. It was found that compounds with *N*-Boc substitution of the benzimidazole exhibit higher inhibitory potency than the corresponding *N*-unsubstituted derivatives and even exceeded the potency of the lead structure diflapolin. Consequently, the Free-Wilson activity contribution for the *N*-Boc substitution on sEH was calculated to be 1.0705. While the sEH activities of the unsubstituted benzimidazoles were weaker compared to those including the *N*-Boc substitution, they also showed improved FLAP activity. In the ADME prediction the four unsubstituted benzimidazoles **6a**, **6b**, **6e,** and **6c** were predicted to have the highest GI absorption rate of the series.

Introduction of the thiourea moiety (compounds **6d**, **6e**, and **6f**) had no significant effect on FLAP activity, in comparison with the urea compound, however, there is a clear decrease in sEH inhibition (Free-Wilson contribution = −1.656). Concerning subunit III, the biological tests showed that the position of the methyl group on the phenylene spacer is not relevant for FLAP activity. Interestingly, the methylation influences the ability to inhibit the sEH. Here, the formal shift of the methyl group from the R’ position next to the urea subunit (**6a**) to the spacer position (**6c**) resulted in a tenfold higher potency. The methyl group receives a low positive contribution in the Free-Wilson analysis on sEH (1.8 × 10^−11^). The absence of the methyl group improves the solubility of the compounds, see Section ADME predictions.

### 2.3. Docking studies

#### 2.3.1. Soluble epoxide hydrolase

**Figure 6.**
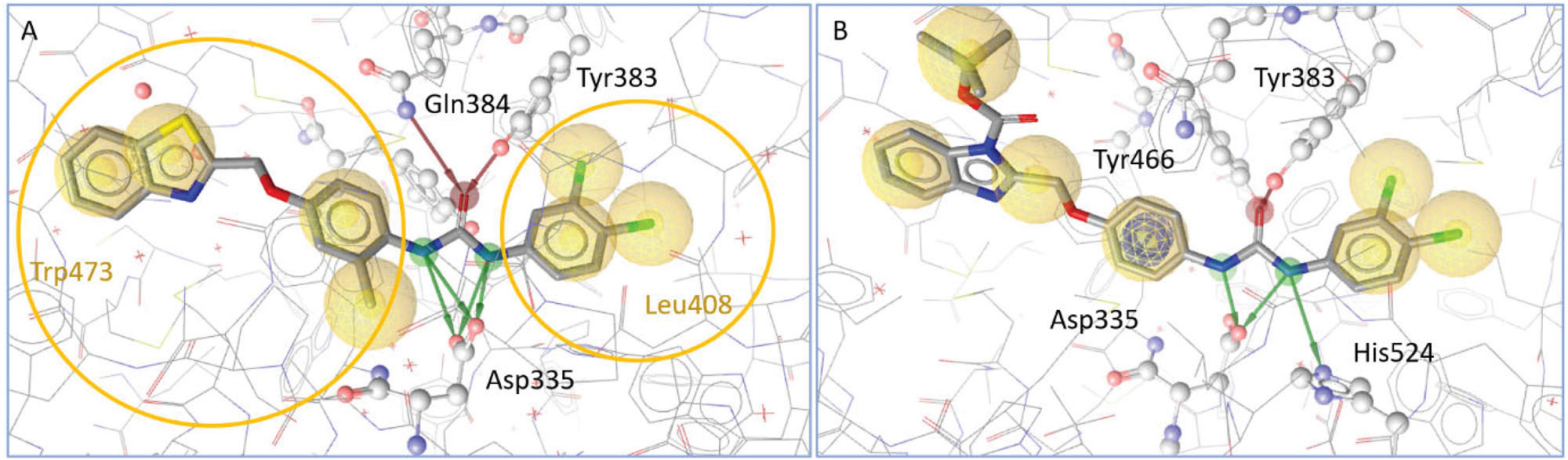
A shows diflapolin within the binding site of sEH. Red arrows signify hydrogen bond acceptor interactions, while green arrows mark hydrogen bond donor interactions. The yellow spheres represent hydrophobic contacts between protein and ligand. The urea moiety of diflapolin is bound to Asp335, Tyr383, Gln384, and in some poses Tyr466 (not shown). The two yellow rings mark the two hydrophobic cavities on both sides of the catalytic triad leading up to Leu408 and Trp473. B shows the most active molecule of the series **5b** bound to sEH with hydrogen bonds to Tyr383, Tyr466, Asp335, and His524.

The active site of sEH is primarily defined by three key amino acids forming the catalytic triad that binds amid related structural motifs. The triad is formed by two tyrosins (Tyr466, Tyr383) and one aspartate (Asp335), which interact via hydrogen bonding with the ligand. In some cases, also hydrogen bonds with His524 and Gln384 are formed. On both sides of these motif hydrophobic cavities accommodate the binding ligands. One pocket is leading up to Trp473, the other one to Leu408. (Figure 6(A)). From the benzimidazole dataset, all active molecules were oriented in the same way within the binding pocket and formed interactions with at least two of the three residues forming the catalytic triad. The most active molecule **5b** (Figure 6(B)) formed hydrogen bonds with four residues in the binding site. The only sEH inactive compound **5d** only formed a single interaction with Tyr466 in the docking simulation. While the docking scores did not correlate quantitatively with bioactivity, the more potent inhibitors formed more of the crucial hydrogen bonds with the catalytic triad. The most potent compounds formed hydrogen bonds with four residues in the binding pocket (see Table 2). The sulphur atom in thiourea moiety did not act as often as a hydrogen bond acceptor as the oxygen atom in the urea containing ligands but the functionality still fit the binding site and formed the key interactions with the nitrogen atoms.

**Table 2.** Surface interactions (SI) of the compounds within the sEH.

Compound	sEH	sEH	sEH	sEH	sEH	sEH number of
	Asp335	Tyr383	Gln384	Tyr466	His524	hydrogen bonds
**Diflapolin**	HBD*	HBA**	HBA			3
**5a**	HBD	HBA		HBA	HBD	4
**5b**	HBD	HBA		HBA	HBD	4
**5c**	HBD	HBA		HBA	HBD	4
**5d**				HBD		1
**5e**		HBA		HBA		2
**5f**	HBD			HBD		2
**6a**		HBA		HBA		2
**6b**	HBD	HBA		HBA		3
**6c**	HBD	HBA		HBA	HBD	4
**6d**	HBD			HBD		2
**6e**	HBD	HBD				2
**6f**				HBD		1

* HBD: hydrogen bond donor.

** HDA: hydrogen bond acceptor.

#### 2.3.2. Five lipoxygenase activating protein

A new crystal structure of FLAP, bound to a dual microsomal prostaglandin E synthase and FLAP inhibitor (PDB code 6VGC^28^) was recently published and used to analyse the compounds binding interaction patterns. To evaluate the docking workflow on the structure, redocking and cross-docking experiments^29^ were conducted and two key interaction points with the binding site were identified: (i) an ionic (or hydrogen bond) interaction with Lys116 was found to be crucial for FLAP inhibition and (ii) interactions with Phe114 or the co-crystallised water molecule 308 (HOH308), which in turn was bound by Phe114 and Tyr90). Detailed settings and docking scores can be found in the results section.

**Figure 7.**
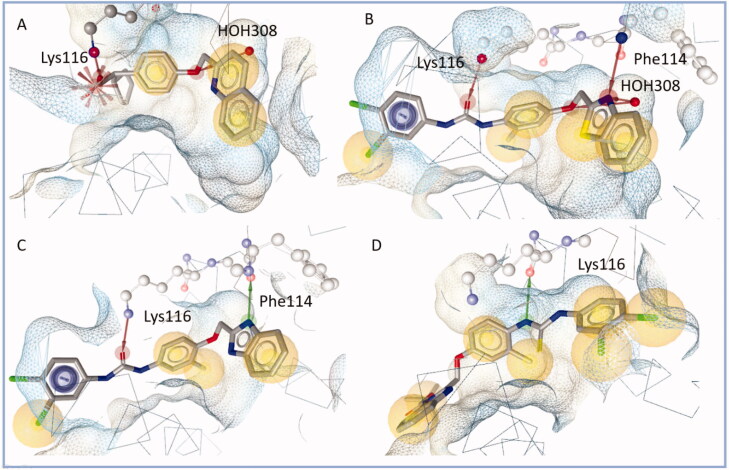
A: Co-crystallised ligand DG-031 within the binding site of FLAP. Blue circles show aromatic interactions. Red stars mark ionic interactions. Red arrows signify hydrogen bond acceptor interactions, while green arrows mark hydrogen bond donor interactions. The yellow spheres represent hydrophobic contacts between protein and ligand. The ligand is anchored with two interactions, the ionic interaction with Lys116 and a hydrogen bond with HOH308, while filling the hydrophobic pocket. B: Diflapolin within the binding site. While diflapolin doesn’t possess an ionic interaction, it is anchored by hydrogen bonds with Lys116 and Phe114. It also forms a hydrogen bond with HOH308 C: This pattern can also be observed in the most active compound of the series **6** (example **6c**), even though the hydrogen bond to HOH308 is not observed in this case D: Inactive compound **5d** by contrast is flipped in the binding site and forms only one hydrogen bond with Lys116. In this orientation interactions between the nitrogen atom of the benzimidazole ring and PHe114 or HOH308 are no longer possible, which could explain the loss of activity.

The co-crystallised ligand DG-031 formed an ionic interaction between its carboxylic function and Lys116 and a hydrogen bond between its ether functionality and HOH308 (Figure 7(A)). In comparison, diflapolin formed hydrogen bonds between the urea moiety and Lys116. The ether group and the nitrogen of the benzothiazole ring form hydrogen bonds with Phe114 and HOH308 (see Figure 7(B) and Table 3).

**Figure 8.**
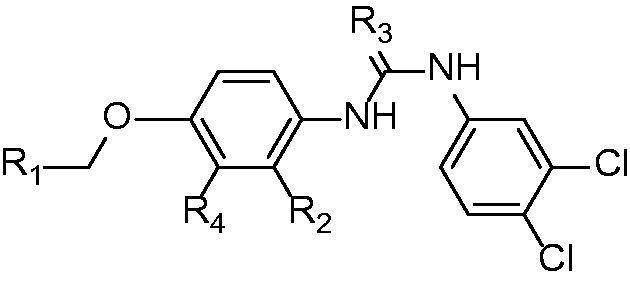
Core molecule for Free-Wilson analysis.

**Figure 9.**
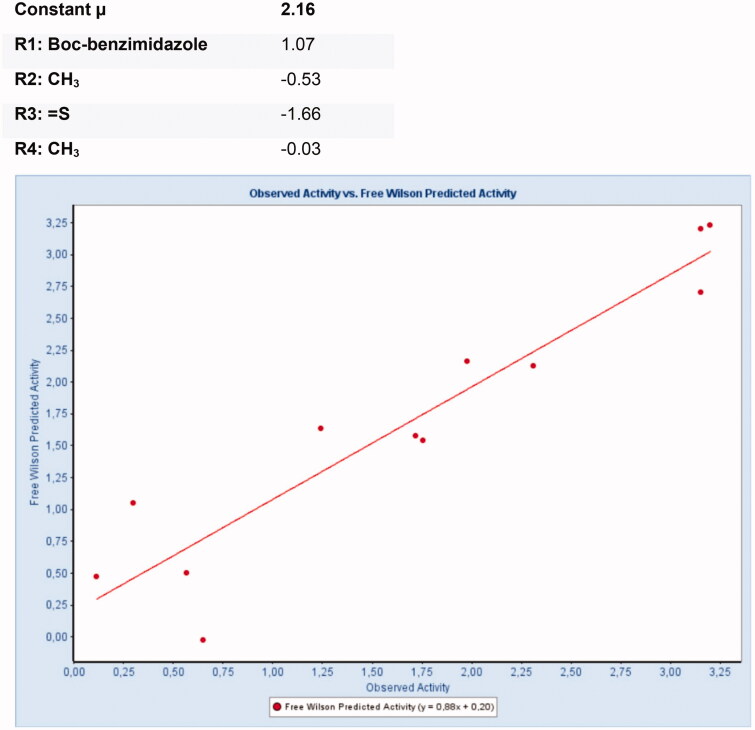
Predicted Free-Wilson activity for sEH vs. observed activity.

**Figure 10.**
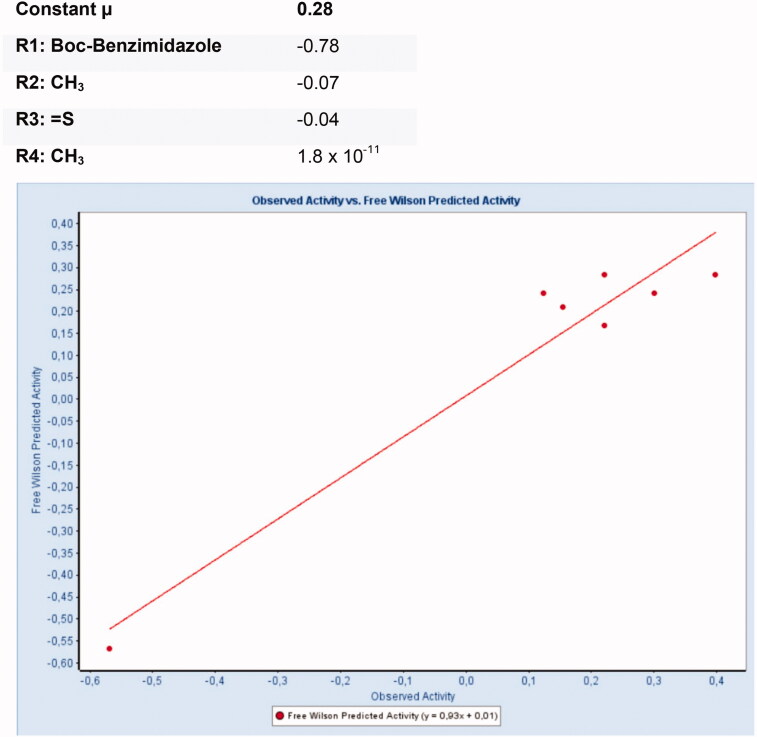
Predicted Free-Wilson activity for FLAP vs. observed activity.

**Table 3.** Surface Interactions (SI) of the compounds within FLAP.

Compound	FLAP	FLAP	FLAP	FLAP	FLAP
	Phe114	Lys116	HOH308	Thr 66	His28
**Diflapolin**	HBA	HBA	HBA	HBA	
**5a**	HBA	HBA	HBA		
**5b**			HBA	HBA	
**5c**			HBA	HBA	
**5d**			HBA	HBA	
**5e**			HBA	HBA	
**5f**				HBA	
**6a**	HBD	HBA			
**6b**	HBD	HBA			
**6c**	HBD	HBA			
**6d**	HBD	HBA			
**6e**	HBD	HBA			HBA
**6f**	HBD	HBA			

The crucial interaction with Lys116 was formed by all active compounds, further stabilised by interactions with Phe114 and/or HOH308 as shown for the most active compound **6c** (Figure 7(C)). In contrast, the inactive compounds **5b–5f** were twisted around within the binding site and only showed either an interaction with Lys116 or with Phe114/HOH308 (Figure 7(D)).

## 3. Conclusion

In summary, the most promising dual inhibitors of these series were **5a**, **6b,** and **6c**. The latter two also showed improved likelihood for GI absorption. Compound **5b** was a very strong selective sEH inhibitor that could be used in future experiments to further elucidate the effects of the dual activity compared to selective inhibition of sEH. The study shows that the benzimidazole retains the dual activity of the benzothiazole.

For sEH activity we can conclude that the urea moiety cannot be exchanged for a thiourea group without loss of activity, though **5e** still represents a potent sEH inhibitor. The *N*-butoxycarbonyl-substitution of the benzimidazole is clearly advantageous for sEH activity and can be used to push for sEH selectivity. Switching the location of the methyl group at the central ring or removing it entirely does not affect sEH activity in case of the *N*-butoxycarbonyl-substituated **5** series but in the unsubstituted **6** series it can be observed that the R position is the most advantageous for sEH activity.

For FLAP activity the *N*-butoxycarbonyl-substitution is clearly obstructing binding, the only exception being compound **5a**, which still binds with an IC_50_ of 3.7 µM. But also, in this case a significant loss compared to unsubstituted **6a** can be observed. Changes to the urea moiety or the orientation of the central ring methyl group do not affect FLAP activity.

For dual activity it can be concluded that FLAP is most sensitive to changes to the heterocyclic system because it requires this part of the molecule for hydrogen bonding with Phe114 and HOH308. sEH activity on the other hand requires the urea moiety for binding to the catalytic triad and profits from bulkier substitution on the benzimidazole ring.

Based on these results, further modifications of the different substructures will be performed.

## 4. Experimental

### 4.1. Chemistry

Reactions were monitored by TLC using Polygram® SIL G/UV254 (Macherey-Nagel) plastic-backed plates (0.25 mm layer thickness), column chromatography was performed using silica gel 60 (40–63 µm). The yields are not optimised. Microwave-assisted reaction was carried out using a PC operated (Synergy software) CEM Discover apparatus, provided with a fibre optic temperature control. Melting points were determined with a Kofler hot-stage microscope (Reichert) and are uncorrected. IR spectra were recorded on a Bruker ALPHA FT-IR apparatus equipped with a Platinum ATR module. ^1^H NMR spectra were recorded on Varian Gemini 200 spectrometer (200 MHz) or “Mars” 400 MHz Bruker Avance 4 Neo spectrometer, ^13^C NMR spectra were recorded on “Mars” 400 MHz Bruker Avance 4 Neo spectrometer with DMSO-d_6_ as the solvent. The centre of the solvent multiplet (DMSO-d_6_) was used as internal standard (chemical shifts in δ ppm), which was related to TMS with δ 2.49 ppm (^1^H) and 39.5 ppm (^13^C). Coupling constants (J) were reported in hertz, and splitting patters were used as follows: s, singlet; br. s, broad singlet; d, doublet; dd, doublet of doublets; m, multiplet. Elemental analyses were performed by Mag. J. Theiner, “Mikroanalytisches Laboratorium,” Faculty of Chemistry, University of Vienna, Austria.

#### 4.1.1. General procedure for the synthesis of compounds 3a-c

A suspension of *tert*-butyl 2-(chloromethyl)-1*H*-benzo[*d*]imidazole-1-carboxylate^25^ (7.5 mmol, 1 equiv.) with 3-methyl-4-nitrophenol, 2-methyl-4-nitrophenol or (9 mmol, 1.2 equiv.) in the presence of potassium carbonate (11 mmol, 1.5 equiv.) in acetone (30 ml) was heated under reflux. The mixture was filtered, and acetone was partially reduced under vacuo. The corresponding product was isolated and purified as described below.

##### 4.1.1.1. *tert*-butyl-2-((3-methyl-4-nitrophenoxy)methyl)-1*H*-benzo[*d*]imidazole-1-carboxylate (3a)

Water was added and the product was extracted with dichloromethane. Then, the organic phase was washed with 2 N NaOH, water, and saturated NaCl solution, dried over Na_2_SO_4_ and evaporated. The pure product was obtained by column chromatography using ethyl acetate as the mobile phase. Yield: 71%; mp 152–154 °C. ^1^H-NMR (200 MHz DMSO-d_6_) δ 8.07–7.95 (m, 2H, Ar–H), 7.71–7.67 (m, 1H, Ar–H), 7.46‐7.31 (m, 2H, Ar–H ), 7.17‐7.07 (m, 2H, Ar–H ), 5.65 (s, 2H, CH_2_), 2.54 (s, 3H, CH_3_), 1.62 (s, 9H, (CH_3_)_3_). IR [cm^−1^] 1735.

##### 4.1.1.2. *tert*-butyl-2-((2-methyl-4-nitrophenoxy)methyl)-1*H*-benzo[*d*]imidazole-1-carboxylate (3 b)

After addition of water, the yellowish solid was collected and dried, giving the pure product. Yield: 84%; 165–168 °C. ^1^H-NMR (200 MHz DMSO-d_6_) δ 8.11–7.96 (m, 3H, Ar–H ), 7.70 (s, *J* = 7.2, 1H, Ar–H ), 7.46–7.28 (m, 3H, Ar–H ), 5.69 (s, 2H, CH_2_), 2.26 (s, 3H, CH_3_), 1.59 (s, 9H, (CH_3_)_3_). IR [cm^−1^] 1753.

##### 4.1.1.3. *tert*-butyl 2-((4-nitrophenoxy)methyl)-1*H*-benzo[*d*]imidazole-1-carboxylate (3c)

A mixture of 4-nitrophenol (2.4 mmol, 1.2 equiv.) and potassium carbonate (3 mmol, 1.5 equiv.) in acetone (2 ml) was stirred at room temperature for 3 h. Then, *tert*-butyl 2-(chloromethyl)-1*H*-benzo[*d*]imidazole-1-carboxylate (2 mmol, 1 equiv.) was added, and the resulting reaction mixture was microwave-assisted heated at 70 °C for 3 h (closed vessel). Water was added and the product was extracted with dichloromethane. Then, the organic phase was washed with 2 N NaOH, water, and saturated NaCl solution, dried over Na_2_SO_4_ and evaporated. The pure product was obtained by column chromatography using ethyl acetate as the mobile phase. Yield: 39%; mp 149–151 °C. ^1^H-NMR (200 MHz DMSO-d_6_) δ 8.23–8.18 (m, 2H, Ar–H ), 7.97 (d, *J* = 8.0 Hz, 1H, Ar–H ), 7.69 (d, *J* = 7.2 Hz, 1H, Ar–H ), 7.46–7.25 (m, 4H, Ar–H), 5.69 (s, 2H, CH_2_), 1.61 (s, 9H, (CH_3_)_3_). IR [cm^−1^] 1750 (C = O).

#### 4.1.2. General procedure for the synthesis of compounds 4a-c

The reduction of the nitro function was done according to literature.^30^ A suspension of the appropriate nitro compounds (5 mmol, 1.equiv), in methanol (60–70 ml) with ammonium formate (15 mmol, 10 equiv.) and palladium on charcoal as catalyst was heated at reflux for two hours (determined by TLC). To remove the catalyst, the resulting mixture was filtered, and the solution was evaporated under reduced pressure.

##### 4.1.2.1. *tert*-butyl 2-((4-amino-3-methylphenoxy)methyl)-1*H*-benzo[*d*]imidazole-1-carboxylate (4a)

The pure product was obtained by column chromatography using ethyl acetate/dichloromethane (2/1) as the mobile phase followed by recrystallisation from isopropanol yielded 84% of light pink crystals, mp 117–118 °C. ^1^H-NMR (200 MHz DMSO-d_6_) δ 7.97–7.94 (m, 1H, Ar–H ), 7.73–7.69 (m, 1H, Ar–H), 7.45–7.31 (m, 2H, Ar–H), 6.66–6.50 (m, 3H, Ar–H), 5.31 (s, 2H, CH_2_), 4.42 (s, 2H, NH_2_, exchangeable with D_2_O), 2.02 (s, 3H, CH_3_), 1.58 (s, 9H, (CH_3_)_3_). IR [cm^−1^] 3314, 3226 (NH_2_), 1737 (C = O).

##### 4.1.2.2. *tert*-butyl 2-((4-amino-2-methylphenoxy)methyl)-1*H*-benzo[*d*]imidazole-1-carboxylate (4 b)

The product thus obtained in 87% yield (brownish solid) was used without further purification. ^1^H-NMR (400 MHz DMSO-d_6_) δ 7.96 (d, *J* = 8.0 Hz, 1H, Ar–H), 7.71 (d, *J* = 7.6 Hz, 1H, Ar–H), 7.43–7.34 (m, 2H, Ar–H) 6.75 (s, *J* = 8.4, 1H, Ar–H), 6.39–6.32 (m, 2H, Ar–H), 5.31 (s, 2H, CH_2_), 4.54 (s, 2H, NH_2_, exchangeable with D_2_O), 2.01 (s, 3H, CH_3_), 1.58 (s, 9H, (CH_3_)_3_). IR [cm^−1^] 3345, 3216 (NH_2_), 1744 (C = O).

##### 4.1.2.3. *tert*-butyl 2-((4-aminophenoxy)methyl)-1*H*-benzo[*d*]imidazole-1-carboxylate (4c)

The pure product was obtained by column chromatography using ethyl acetate/dichloromethane (2/1) as the mobile phase yielded 76% of light pink crystals, mp 100–101 °C. ^1^H-NMR (200 MHz DMSO-d_6_) δ 7.98–7.94 (m, 1H, Ar–H), 7.73–7.69 (m, 1H, Ar–H), 7.44–7.31 (m, 2H, Ar–H), 6.73 (d, *J* = 8.8 Hz, 2H, Ar–H), 6.50 (d, J = 8.8 Hz, 2H, Ar–H), 5.31 (s, 2H, CH_2_), 4.65 (s, 2H, NH_2_ exchangeable with D_2_O), 1.58 (s, 9H, (CH_3_)_3_). IR [cm^−1^] 3343, 3221, 1741.

#### 4.1.3. General procedure for the synthesis of compounds 5a-f

To a solution of the amino compound (1 equiv.) in tetrahydrofuran (5 ml) was added dropwise a solution of 3,4-dichlorophenylisocyanate or 3,4-dichlorophenylisothiocyanate (1.2 equiv.) in tetrahydrofuran. The resulting mixture was stirred until the amino compound was completely consumed. The crystals thus obtained were collected by filtration, washed with diethyl ether and were purified as described below.

##### 4.1.3.1. *tert*-butyl-2-((4-(3-(3,4-dichlorophenyl)ureido)-3-methylphenoxy)methyl)-1*H*-benzo[*d*]imidazole-1-carboxylate (5a)

Recrystallisation from ethanol yielded 69% of a colourless powder, mp 261–263 °C. ^1^H-NMR (400 MHz DMSO-d_6_) 9.13 (s, 1H, NH, exchangeable with D_2_O), 7.97 (d, *J* = 8.0 Hz, 1H, Ar–H), 7.91 (s, 1H, NH, exchangeable with D_2_O), 7.88 (d, *J* = 2.8 Hz, 1H, Ar–H), 7.71 (d, *J* = 7.6 Hz, 1H, Ar–H), 7.48 (d, *J* = 8.8 Hz, 2H, Ar–H), 7.43–7.39 (m, 1H, Ar–H), 7.37–7.33 (m, 1H, Ar–H), 7.28 (dd, *J* = 8.8 Hz, *J* = 2.8 Hz, 1H, Ar–H), 6.90 (d, *J* = 2.8 Hz, 1H, Ar–H), 6.84 (dd, *J* = 8.8 Hz, *J* = 2.8 Hz, 1H, Ar–H), 5.45 (s, 2H, CH_2_), 2.20 (s, 3H, CH_3_), 1.61 (s, 9H, (CH_3_)_3_). ^13 ^C-NMR (100 MHz DMSO-d_6_) 154.5, 152.8, 150.6, 147.8, 141.5, 140.3, 132.9, 131.6, 131.0, 130.5, 130.1, 125.1, 124.5, 124.2, 122.7, 119.9, 119.0, 118.0, 116.2, 114.6, 111.9, 85.8, 64.6, 27.4, 18.0. IR [cm^−1^] 3356, 1742, 1702. Anal. Calcd for: C_27_H_26_Cl_2_N_4_O_4_ x 0.8 H_2_O (555.85): C, 58.34; H, 5.00; N, 10.00; Found: C, 58.33; H, 4.98; N, 9.97.

##### 4.1.3.2. *tert*-butyl 2-((4-(3-(3,4-dichlorophenyl)ureido)phenoxy)methyl)-1*H*-benzo[*d*]imidazole-1-carboxylate (5b)

Recrystallisation from ethyl acetate yielded 61% of a colourless powder, mp 262–264 °C. ^1^H-NMR (400 MHz DMSO-d_6_) 8.91 (s, 1H, NH, exchangeable with D_2_O), 8.62 (s, 1H, NH, exchangeable with D_2_O), 7.97 (d, *J* = 7.4 Hz, 1H, Ar–H), 7.86 (d, *J* = 2.4 Hz, 1H, Ar–H), 7.71 (d, *J* = 7.4 Hz, 1H, Ar–H), 7.49 (d, *J* = 8.8 Hz, 1H, Ar–H), 7.44–7.39 (m, 1H, Ar–H), 7.38–7.33 (m, 1H, Ar–H), 7.35 (d, *J* = 9.2 Hz, 2H, Ar–H), 7.30 (dd, *J* = 8.8 Hz, *J* = 2.4 Hz, 1H, Ar–H), 6.98 (d, *J* = 9.2 Hz, 2H, Ar–H), 5.45 (s, 2H, CH_2_), 1.59 (s, 9H, (CH_3_)_3_). ^13 ^C-NMR (100 MHz DMSO-d_6_) 153.5, 152.4, 150.6, 147.8, 141.5, 140.1, 132.9, 132.7, 131.0, 130.5, 125.1, 124.2, 122.8, 120.3, 119.9, 119.1, 118.2, 114.8, 114.6, 85.8, 64.7, 27.4. IR [cm^−1^] 1749, 1708. Anal. Calcd for: C_26_H_24_Cl_2_N_4_O_4_ (527,41): C, 59.21; H, 4.59; N, 10.62; Found: C, 59.08; H, 4.64; N, 10.38.

##### 4.1.3.3. *tert*-butyl-2-((4-(3-(3,4-dichlorophenyl)ureido)-2-methylphenoxy)methyl)-1*H*-benzo[*d*]imidazole-1-carboxylate (5c)

Column chromatography with dichloromethane/ethyl acetate (2/1) as eluent followed by recrystallisation from acetone yielded 15% of a colourless powder, mp 145–146 °C. ^1^H-NMR (400 MHz DMSO-d_6_) 8.89 (s, 1H, NH, exchangeable with D_2_O), 8.53 (s, 1H, NH, exchangeable with D_2_O), 7.97 (d, *J* = 8.0 Hz, 1H, Ar–H), 7.87 (d, *J* = 2.4 Hz, 1H, Ar–H), 7.72 (d, *J* = 7.6 Hz, 1H, Ar–H), 7.48 (d, *J* = 8.8 Hz, 1H, Ar–H), 7.44–7.40 (m, 1H, Ar–H), 7.38–7.34 (m, 1H, Ar–H), 7.29 (dd, *J* = 8.8 Hz, *J* = 2.4 Hz, 1H, Ar–H), 7.24 (d, *J* = 2.4 Hz, 1H, Ar–H), 7.19 (dd, *J* = 8.8 Hz, *J* = 2.4 Hz, 1H, Ar–H), 7.00 (d, *J* = 8.8 Hz, 1H, Ar–H), 5.45 (s, 2H, CH_2_), 2.13 (s, 3H, CH_3_), 1.58 (s, 9H, (CH_3_)_3_). ^13 ^C-NMR (100 MHz DMSO-d_6_) 152.4, 151.8, 150.7, 147.8, 141.5, 140.2, 133.0, 132.3, 131.0, 130.5, 126.0, 125.2, 124.3, 122.8, 121.7, 120.0, 119.1, 118.2, 117.4, 114.7, 112.1, 85.8, 65.0, 27.4, 16.2. IR [cm^−1^] 3463, 1753, 1701. Anal. Calcd for C_27_H_26_Cl_2_N_4_O_4_ (541.43): C, 59.90; H, 4.84; N, 10.35; Found: C, 59.62; H, 4.51; N, 10.46.

##### 4.1.3.4. *tert*-butyl 2-((4-(3-(3,4-dichlorophenyl)thioureido)-3-methylphenoxy)methyl)-1*H*-benzo[*d*]imidazole-1-carboxylate (5d)

Column chromatography with dichloromethane/ethyl acetate (5/1) to tetrahydrofuran as eluent followed by recrystallisation from ethanol/tetrahydrofuran yielded 76% of a colourless powder, mp 172 °C. ^1^H-NMR (400 MHz DMSO-d_6_) 9.70 (br. s, 1H, NH, exchangeable with D_2_O), 9.45 (s, 1H, NH, exchangeable with D_2_O), 7.97 (d, *J* = 8.0 Hz, 1H, Ar–H), 7.89–7.88 (m, 1H, Ar–H), 7.72 (d, *J* = 7.6 Hz, 1H, Ar–H), 7.54 (d, *J* = 8.8 Hz, 1H, Ar–H), 7.46–7.39 (m, 2H, Ar–H), 7.37–7.33 (m, 1H, Ar–H), 7.12 (d, *J* = 8.4 Hz, 1H, Ar–H), 6.95 (d, *J* = 2.8 Hz, 1H, Ar–H), 6.87 (dd, *J* = 8.4 Hz, *J* = 2.8 Hz, 1H, Ar–H), 5.49 (s, 2H, CH_2_), 2.19 (s, 3H, CH_3_), 1.62 (s, 9H, (CH_3_)_3_). ^13 ^C-NMR (100 MHz DMSO-d_6_) 180.7, 156.7, 150.6, 147.8, 141.5, 139.9, 136.6, 132.8, 130.6, 130.3, 130.0, 129.2, 125.7, 125.1, 124.9, 124.2, 123.7, 119.9, 116.2, 114.7, 112.2, 85.9, 64.6, 27.4, 18.0. IR [cm^−1^] 3180, 3350, 1749. Anal. Calcd for: C_27_H_26_Cl_2_N_4_O_3_S (557.50): C, 58.17; H, 4.70; N, 10.05; S, 5.75; Found: C, 58.19; H, 4.68; N, 10.00; S, 5.71.

##### 4.1.3.5. *tert*-butyl 2-((4-(3-(3,4-dichlorophenyl)thioureido)phenoxy)methyl)-1*H*-benzo[*d*]imidazole-1-carboxylate (5e)

Column chromatography with dichloromethane/ethyl acetate (5/1) as eluent followed by recrystallisation from ethyl acetate yielded 61% of a colourless powder, mp 165–167 °C. ^1^H-NMR (400 MHz DMSO-d_6_) 9.84 (s, 1H, NH, exchangeable with D_2_O), 9.82 (s, 1H, NH, exchangeable with D_2_O), 7.97 (d, *J* = 8.0 Hz, 1H, Ar–H), 7.87 (d, *J* = 2.4 Hz, 1H, Ar–H), 7.72 (d, *J* = 7.6 Hz, 1H, Ar–H), 7.54 (d, *J* = 8.8 Hz, 1H, Ar–H), 7.44–7.32 (m, 5H, Ar–H), 7.03 (d, *J* = 9.2 Hz, 2H, Ar–H), 5.49 (s, 2H, CH_2_), 1.61 (s, 9H, (CH_3_)_3_). ^13 ^C-NMR (100 MHz DMSO-d_6_) 179.8, 155.6, 150.5, 147.9, 141.5, 139.9, 132.9, 132.2, 130.4, 130.1, 126.0, 125.8, 125.2, 124.8, 124.3, 123.5, 119.9, 114.7, 114.5, 85.9, 64.7, 27.4. IR [cm^−1^] 3154, 3363, 1751. Anal. Calcd for: C_26_H_24_Cl_2_N_4_O_3_S (543.47): C, 57.46; H, 4.45; N, 10.31; S, 5.90; Found: C, 57.45; H, 4.45; N, 10.26; S, 5.86.

##### 4.1.3.6. *tert*-butyl 2-((4–(3-(3,4-dichlorophenyl)thioureido)-2-methylphenoxy)methyl)-1*H*-benzo[*d*]imidazole-1-carboxylate (5f)

Column chromatography with dichloromethane/ethyl acetate (5/1) as eluent followed by recrystallisation from isopropanol yielded 59% of a colourless powder, mp 169–172 °C. ^1^H-NMR (400 MHz DMSO-d_6_) 9.78 (br. s, 2H, NH, exchangeable with D_2_O), 7.97 (d, *J* = 8.0 Hz, 1H, Ar–H), 7.86 (d, *J* = 2.4 Hz, 1H, Ar–H), 7.73 (d, *J* = 7.6 Hz, 1H, A-rH), 7.54 (d, *J* = 8.4 Hz, 1H, Ar–H), 7.43–7.34 (m, 3H, Ar–H), 7.18–7.15 (m, 2H, Ar–H), 7.05 (d, *J* = 8.4 Hz, 1H, Ar–H), 5.50 (s, 2H, CH_2_), 2.15 (s, 3H, CH_3_), 1.60 (s, 9H, (CH_3_)_3_). ^13 ^C-NMR (100 MHz DMSO-d_6_) 179.7, 153.8, 150.6, 147.8, 141.5, 140.0, 132.9, 131.7, 130.3, 130.0, 127.1, 125.9, 125.7, 125.2, 124.9, 124.3, 123.6, 123.3, 120.0, 114.7, 111.7, 85.9, 64.9, 27.4, 16.1. IR [cm^−1^] 3361, 1747. Anal. Calcd for: C_27_H_26_Cl_2_N_4_O_3_S (557.50): C, 58.17; H, 4.70; N, 10.05; S, 5.75; Found: C, 58.11; H, 4.65; N, 9.84; S, 5.59.

#### 4.1.4. General procedure for the synthesis of compounds 6a-f

The elimination of the protecting group was done according to literature.^31^ A suspension of the protected compounds (1 mmol, 1 equiv.) and trifluoroacetic acid (10 mmol, 10 equiv.) was heated at 30 °C until the reaction was completed. The mixture was cooled, neutralised with saturated sodium bicarbonate solution and the product was extracted with ethyl acetate. The organic layer was washed with water and brine, dried over sodium sulphate, and evaporated *in vacuo*. The products thus obtained were purified as described below.

##### 4.1.4.1. 1-(4-((1*H*-benzo[*d*]imidazol-2-yl)methoxy)-2-methylphenyl)-3-(3,4-dichlorophenyl)urea (6a)

Recrystallisation from 1,4-dioxane yielded 35% of a colourless powder, mp 245–246 °C. ^1^H-NMR (400 MHz DMSO-d_6_) 12.63 (s, 1H, NH, exchangeable with D_2_O), 9.14 (s, 1H, NH, exchangeable with D_2_O), 7.92 (s, 1H, NH, exchangeable with D_2_O), 7.87 (s, 1H, Ar–H), 7.60 (d, *J* = 7.6 Hz, 1H, Ar–H), 7.49–7.47 (m, 3H, Ar–H), 7.28 (d, *J* = 8.0 Hz, 1H, Ar–H), 7.21–7.16 (m, 2H, Ar–H), 6.95 (s, 1H, Ar–H), 6.89 (d, *J* = 8.8 Hz, 1H, Ar–H), 5.26 (s, 2H, CH_2_), 2.19 (s, 3H, CH_3_). ^13 ^C-NMR (100 MHz DMSO-d_6_) 154.5, 152.9, 150.2, 140.3, 131.7, 131.0, 130.6, 130.4, 124.5, 122.8, 122.0, 119.0, 118.1, 116.5, 112.363.9, 18.1. IR [cm^−1^] 3278, 1639. Anal. Calcd for: C_22_H_18_Cl_2_N_4_O_4_ x 0.6 H_2_O (452.13): C, 58.44; H, 4.28; N, 12.39; Found: C, 58.57; H, 4.07; N, 12.06.

##### 4.1.4.2. 1-(4-((1*H*-benzo[*d*]imidazol-2-yl)methoxy)phenyl)-3-(3,4-dichlorophenyl)urea (6b)

Column chromatography with dichloromethane/tetrahydrofuran (5/1 to 0/1) as eluent followed by recrystallisation from isopropanol yielded 31% of a colourless powder, mp 272 °C. ^1^H-NMR (400 MHz DMSO-d_6_) 12.64 (s, 1H, NH, exchangeable with D_2_O), 8.92 (s, 1H, NH, exchangeable with D_2_O), 8.63 (s, 1H, NH, exchangeable with D_2_O), 7.85 (d, *J* = 2.4 Hz, 1H, Ar–H), 7.60 (d, *J* = 7.6 Hz, 1H, Ar–H), 7.48 (d, *J* = 8.8 Hz, 1H, Ar–H), 7.47 (d, *J* = 7.6 Hz, 1H, Ar–H), 7.36 (d, *J* = 7.0 Hz, 2H, Ar–H), 7.30 (dd, *J* = 8.8 Hz, *J* = 2.4 Hz, 1H, Ar–H), 7.21–7.14 (m, 2H, Ar–H), 7.03 (d, *J* = 7.0 Hz, 2H, Ar–H), 5.26 (s, 2H, CH_2_). ^13 ^C-NMR (100 MHz DMSO-d_6_) 153.4, 152.5, 150.3, 142.9, 140.1, 134.3, 132.9, 131.0, 130.5, 122.9, 122.4, 121.4, 120.4, 119.2, 118.9, 118.3, 115.1, 111.5, 64.1. IR [cm^−1^] 3277, 1636. Anal. Calcd for: C_21_H_16_Cl_2_N_4_O_2_ x 0.2 H_2_O (430.90): C, 58.54; H, 3.84; N, 13.00; Found: C, 58.64; H, 3.72; N, 12.56.

##### 4.1.4.3. 1-(4-((1*H*-benzo[*d*]imidazol-2-yl)methoxy)-3-methylphenyl)-3-(3,4-dichlorophenyl)urea (6c)

Column chromatography with dichloromethane/tetrahydrofuran (5/1 to 0/1) as eluent followed by recrystallisation from isopropanol yielded 55% of a colourless powder, mp 228–230 °C. ^1^H-NMR (400 MHz DMSO-d_6_) 12.66 (s, 1H, NH, exchangeable with D_2_O), 10.02 (s, 1H, NH, exchangeable with D_2_O), 9.38 (s, 1H, NH, exchangeable with D_2_O), 7.86 (d, *J* = 2.4 Hz, 1H, Ar–H), 7.65–7.45 (m, 2H, Ar–H), 7.47 (d, *J* = 8.4 Hz, 1H, Ar–H), 7.30 (dd, *J* = 8.8 Hz, *J* = 2.4 Hz, 1H, Ar–H), 7.23–7.17 (m, 4H, Ar–H), 7.02 (d, *J* = 8.4 Hz, 1H, Ar–H), 5.25 (s, 2H, CH_2_), 2.20 (s, 3H, CH_3_). ^13 ^C-NMR (100 MHz DMSO-d_6_) 152.7, 151.4, 150.3, 140.5, 132.9, 131.0, 130.5, 126.5, 122.4, 121.3, 121.1, 118.8, 118.5, 117.7, 116.7, 112.4, 64.3, 16.3. IR [cm^−1^] 3290, 1637. Anal. Calcd for: C_22_H_18_Cl_2_N_4_O_2_ x 0.5 H_2_O (450.33): C, 58.68; H, 4.25; N, 12.44; Found: C, 58.69; H, 3.90; N, 12.14.

##### 4.1.4.4. 1-(4-((1*H*-benzo[*d*]imidazol-2-yl)methoxy)-2-methylphenyl)-3-(3,4-dichlorophenyl)thiourea (6d)

Column chromatography with dichloromethane/tetrahydrofuran (7/1) as eluent followed by recrystallisation from methanol yielded 39% of a colourless powder, mp 122–124 °C. ^1^H-NMR (400 MHz DMSO-d_6_) 12.66 (s, 1H, NH, exchangeable with D_2_O), 9.70 (br. s, 1H, NH, exchangeable with D_2_O), 9.46 (s, 1H, NH, exchangeable with D_2_O), 7.88 (d, *J* = 2.0 Hz, 1H, Ar–H), 7.61 (d, *J* = 8.0 Hz, 1H, Ar–H), 7.54 (d, *J* = 8.4 Hz, 1H, Ar–H), 7.48 (d, *J* = 7.2 Hz, 1H, Ar–H), 7.44 (dd, *J* = 8.8 Hz, *J* = 2.4 Hz, 1H, Ar–H), 7.22–7.16 (m, 2H, Ar–H), 7.13 (d, *J* = 8.8 Hz, 1H, Ar–H), 7.00 (d, *J* = 2.8 Hz, 1H, Ar–H), 6.92 (dd, *J* = 8.8 Hz, *J* = 2.8 Hz, 1H, Ar–H), 5.30 (s, 2H, CH_2_), 2.19 (s, 3H, CH_3_). ^13 ^C-NMR (100 MHz DMSO-d_6_) 180.7, 156.6, 150.0, 142.9, 139.9, 136.7, 134.3, 130.8, 130.3, 130.0, 129.3, 125.8, 124.9, 123.7, 122.4, 121.4, 118.9, 116.4, 112.4, 111.5, 63.9, 18.0. IR no characteristic band. Anal. Calcd for: C_22_H_18_Cl_2_N_4_OS (457.40): C, 57.77; H, 3.97; N, 12.25; S, 7.01; Found: C, 57.41; H, 3.88; N, 12.03; S, 6.97.

##### 4.1.4.5. 1-(4-((1*H*-benzo[*d*]imidazol-2-yl)methoxy)phenyl)-3-(3,4-dichlorophenyl)thiourea (6e)

Column chromatography with dichloromethane/tetrahydrofuran (7/1) as eluent followed by recrystallisation from dichlormethane/diisopropyl ether yielded 43% of a colourless powder, mp 160–162 °C. ^1^H-NMR (400 MHz DMSO-d_6_) 12.68 (br. s, 1H, NH, exchangeable with D_2_O), 9.86 (s, 1H, NH, exchangeable with D_2_O), 9.83 (s, 1H, NH, exchangeable with D_2_O), 7.89 (d, *J* = 2.4 Hz, 1H, Ar–H), 7.57–7.53 (m, 3H, Ar–H), 7.44 (dd, *J* = 8.8 Hz, *J* = 2.4 Hz, 1H, Ar–H), 7.35 (d, *J* = 8.8 Hz, 2H, Ar–H), 7.20–7.16 (m, 2H, Ar–H), 7.08 (d, *J* = 8.8 Hz, 2H, Ar–H), 5.32 (s, 2H, CH_2_). ^13 ^C-NMR (100 MHz DMSO-d_6_) 179.8, 155.4, 150.1, 139.9, 132.4, 130.4, 130.1, 126.0, 125.8, 124.8, 123.6, 121.9, 114.8, 64.0. IR no characteristic band. Anal. Calcd for: C_21_H_16_Cl_2_N_4_OS (443.36): C, 56.89; H, 3.64; N, 12.64, S, 7.23; Found: C, 56.96; H, 3.61; N, 12.30, S, 7.05.

##### 4.1.4.6. 1-(4-((1*H*-benzo[*d*]imidazol-2-yl)methoxy)-3-methylphenyl)-3-(3,4-dichlorophenyl)thiourea (6f)

Column chromatography with ethyl acetate/tetrahydrofuran (7/1) as eluent followed by recrystallisation from tetrahydrofuran yielded 55% of a colourless powder, mp 167–168 °C (mod 133–134 °C). ^1^H-NMR (400 MHz DMSO-d_6_) 12.59 (s, 1H, NH, exchangeable with D_2_O), 9.78 (s, 2H, NH, exchangeable with D_2_O), 7.87 (d, *J* = 2.4 Hz, 1H, Ar–H), 7.63–7.48 (m, 3H, Ar–H), 7.42 (dd, *J* = 8.8 Hz, *J* = 2.4 Hz, 1H, Ar–H), 7.19–7.14 (m, 4H, Ar–H), 7.08 (d, *J* = 8.8 Hz, 1H, Ar–H), 5.32 (s, 2H, CH_2_), 2.22 (s, 3H, CH_3_). ^13 ^C-NMR (100 MHz DMSO-d_6_) 179.7, 153.7, 150.2, 139.9, 131.9, 130.3, 130.0, 127.2, 126.4, 125.7, 124.9, 123.6, 123.3, 122.4, 121.4, 118.9, 111.7, 111.5, 64.2, 16.2. IR no characteristic band. Anal. Calcd for: C_22_H_18_Cl_2_N_4_OS x 0.8 THF x 0.2 H_2_O (518.67): C, 58.36; H, 4.82; N, 10.80; S, 6.18; Found: C, 58.32; H, 4.58; N, 10.80; S, 6.06.

### 4.2. Biological evaluation

#### 4.2.1. Materials and methods

Bovine serum albumin (BSA) and Tris were obtained from AppliChem (Darmstadt, Germany); PGB1, 3-phenyl-cyano(6-methoxy-2-naphthalenyl)methylester-2-oxiraneacetic acid (PHOME) and MK886 from Cayman Chemical (Biomol, Hamburg, Germany); DMSO from Merck (Darmstadt, Germany); zileuton from Sequoia Research Products (Oxford, UK); Dulbecco’s Buffer Substance (PBS) from SERVA Electrophoresis (Heidelberg, Germany); HPLC solvents were from VWR (Darmstadt, Germany); Ca^2+^-ionophore A23187, dextran, and all other chemicals were from Sigma-Aldrich (Taufkirchen, Germany), unless stated elsewhere.

#### 4.2.2. Determination of 5-LOX product formation

Human neutrophils were isolated from buffy coats of healthy adult fasted donors as described recently.^32^ The use of human blood preparations was approved by the ethical commission of the Friedrich Schiller University Jena (approval number 4292–12/14) in accordance with relevant guidelines and regulations. Freshly isolated neutrophils were diluted with Dulbecco’s PBS containing 0.1% glucose and 1 mM CaCl_2_ to a final density of 5 × 10^6^ cells/mL and pre-incubated for 10 min at 37 °C with test compounds or 0.1% vehicle (DMSO), respectively. Cells were subsequently stimulated with 2.5 µM A23187 for 10 min at 37 °C and reactions were terminated using one volume of ice-cold methanol. 200 ng of internal PGB1 standard was added to the acidified samples and solid-phase-extraction using C18 RP-columns (100 mg, UCT, Bristol, PA, USA) was performed. Samples were eluted with methanol and 5-LOX products including 5-HpETE, and the corresponding alcohol 5-HETE, LTB_4_, and all-trans-isomers of LTB_4_ were analysed by RP-HPLC with a C-18 Radial-PAK column (Waters, Eschborn, Germany) as described.^33^

#### 4.2.3. Determination of sEH activity

Human recombinant sEH was expressed and purified as published.^23^ Isolated sEH was diluted in 25 mM Tris buffer (pH 7) containing 0.1 mg/mL BSA to a final protein concentration of 0.5 µg/mL. Preincubation with test compounds or 0.1% vehicle (DMSO) at room temperature for 10 min was followed by stimulation with 50 µM PHOME at RT for 60 min. Reactions were stopped with 200 mM ZnSO_4_ and fluorescence was subsequently detected at λ_em_ 465 nm and λ_ex_ 330 nm. If required, possible fluorescence of the compounds was subtracted from the read-out.

### 4.3. Docking studies

Ligands were prepared by energetically minimising them in Omega 2.5.1.4^34^ (OpenEye Scientific Software, Santa Fe, NM. http://www.eyesopen.com) prior to docking. Docking simulations were performed on a windows 10 machine with an i5CPU using the CCDC’s Gold software (version 5.2).^35^ GOLD was set to calculate ten poses per molecule. If the genetic algorithm leads to very similar poses, this a sign of a stable energetic minimum. The results are shown in Table 4.

**Table 4.** Docking scores.

Compound	R	R′	X	FLAP	sEH
				ChemScore	GoldScore
**Diflapolin**				34.98	79.78
**5a**	H	Me	O	31.74	85.85
**5b**	H	H	O	31.43	92.57
**5c**	Me	H	O	31.76	86.58
**5d**	H	Me	S	30.85	89.46
**5e**	H	H	S	30.34	92.95
**5f**	Me	H	S	33.64	95.70
**6a**	H	Me	O	34.59	83.43
**6b**	H	H	O	33.49	81.19
**6c**	Me	H	O	35.16	81.26
**6d**	H	Me	S	33.58	82.82
**6e**	H	H	S	33.16	82.99
**6f**	Me	H	S	34.10	83.33

For sEH the PDB entry 6hgv^28^ was selected as crystal structure for docking. The bound inhibitor (R)-talinolol was extracted from the structure and its binding site was used to define the docking site in a 6 Å radius. GoldScore, Chemscore, and ChemPLP Score were tried in the redocking. GoldScore was selected as a scoring function because it led to the best replication of the co-crystallised pose. The original ligand was prepared in the same workflow as the candidate molecules and redocked into the binding site, with an RMSD of 1.563.

For FLAP the recently published pdb entry 6vgc^28^ with a resolution of 2.37 Å, co-crystallised with (2 R)-cyclopentyl{4-[(quinolin-2-yl)methoxy]phenyl}acetic acid (DG-031) was selected (A chain). Redocking was performed to find optimal docking settings. A 6 Å radius around the co-crystallised ligand was defined as the binding site. Again GoldScore, Chemscore and ChemPLP Score were evaluated for their performance in the redocking. In this case ChemScore performed best and was selected as a scoring function—leading to an RMSD of the best ranked redocked pose to the co-crystallised ligand of 1.069. To further evaluate the docking workflow the co-crystallised ligand of PDB entry 6vgi, MK-866 was docked into 6vgc. Key interactions from the crystal structure were successfully reproduced. The interactions between the docking poses and the binding site were calculated and visualised in Ligandscout 4.2. (www.inteligand.com).

### 4.4. Docking results

While no correlation between the docking scores and experimental bioactivity could be observed, several key interactions served as powerful indicators of activity.

### 4.5. Free-Wilson analysis

Free-Wilson analysis is a QSAR method to assign to each occurring group in a SAR dataset a contribution to the activity of the molecule. Following the equation

Log  BAi =∑ ajk + Xjk + µ

BAi, the biological activity of a series (on a logarithmic scale) is expressed as the sum of the biological activity contributions ajk of the substituents Rk in each position j, µ is referring to the overall average activity value for the series.^36^

A Free-Wilson least squares model was calculated in Biovia’s Pipeline Pilot (version 2019, Dassault systems, San Diego, CA, USA), using the implemented script “Create Free-Wilson least squares model”. The Free-Wilson predicted activity was based on the pIC_50_ values of the measured compounds.

#### 4.5.1. Free-Wilson analysis for sEH

The Free-Wilson analysis with the pIC_50_ values for sEH activity led to a model with an RMSE of 0.14 and an R^2^ of 0.88 (see Figures 8 and 9). For the individual groups, the following contributions were calculated:

For sEH activity the model showed that the *N*-butoxycarbonyl-substitution of the benzimidazole is highly beneficial for the activity, leading to an activity contribution of 1.70705 for this modification. The introduction of the thiourea moyiety significantly decreased sEH activity (Free-Wilson contribution = −1.66). The methyl group in R4 position is favourable for sEH activity.

#### 4.5.2. Free-Wilson analysis for FLAP

The Free-Wilson analysis with the pIC_50_ values for FLAP activity resulted in a model with an RMSE 0.006 and an R2 of 0.93 (see Figures 8 and 10). For the individual groups, the following contributions were calculated:

For FLAP activity, the model showed that the *N*-butoxycarbonyl-substitution of the benzimidazole damages activity the most with a high negative contribution to the overall activity (-0.78). Other substituents had only a minor contribution to the overall activity. Due to the many inactives in this dataset the relevance of this analysis is limited.

#### 4.5.3. ADME predictions

ADME predictions (see Table 5) were conducted with the SwissADME webtool.^37–39^ These predictions were conducted to gain an insight into the solubility and bioavailability properties of the derivatives. Diflapolin itself has limited solubility and is therefore not easily absorbed. We aim to improve bioavailability for the derivatives to make the structure more suitable for pharmaceutical application.

**Table 5.** Results of the SwissADME prediction.

	GI absorption	BBB permeant	Pgp substrate	CYP1A2 inhibitor	CYP2C19 inhibitor	CYP2C9 inhibitor	CYP2D6 inhibitor	CYP3A4 inhibitor	Lipinski #violations	Bioavailability Score	PAINS #alerts
**5a**	Low	No	No	No	Yes	Yes	Yes	Yes	2	0.17	0
**5b**	Low	No	No	No	Yes	Yes	No	Yes	2	0.17	0
**5c**	Low	No	No	No	Yes	Yes	Yes	Yes	2	0.17	0
**5d**	Low	No	Yes	No	Yes	Yes	Yes	Yes	2	0.17	0
**5e**	Low	No	No	No	Yes	Yes	Yes	Yes	2	0.17	0
**5f**	Low	No	Yes	No	Yes	Yes	Yes	Yes	2	0.17	0
**6a**	High	No	No	Yes	Yes	Yes	Yes	Yes	0	0.55	0
**6b**	High	No	No	Yes	Yes	Yes	Yes	Yes	0	0.55	0
**6c**	High	No	No	Yes	Yes	Yes	Yes	Yes	0	0.55	0
**6d**	Low	No	No	Yes	Yes	Yes	Yes	Yes	0	0.55	0
**6e**	High	No	No	Yes	Yes	Yes	Yes	Yes	0	0.55	0
**6f**	Low	No	No	Yes	Yes	Yes	Yes	Yes	0	0.55	0
**Diflapolin**	Low	No	No	Yes	Yes	Yes	Yes	Yes	1	0.55	0

(*Continued*)
